# Predicting Asthma Using Clinical Indexes

**DOI:** 10.3389/fped.2019.00320

**Published:** 2019-07-31

**Authors:** Jose A. Castro-Rodriguez, Lorena Cifuentes, Fernando D. Martinez

**Affiliations:** ^1^Division of Pediatrics, School of Medicine, Pontificia Universidad Catolica de Chile, Santiago, Chile; ^2^Asthma and Airway Disease Research Center, University of Arizona, Tucson, AZ, United States

**Keywords:** asthma, asthma predictive index, biomarkers, preschool, predictive models, recurrent wheezing

## Abstract

Asthma is no longer considered a single disease, but a common label for a set of heterogeneous conditions with shared clinical symptoms but associated with different cellular and molecular mechanisms. Several wheezing phenotypes coexist at preschool age but not all preschoolers with recurrent wheezing develop asthma at school-age; and since at the present no accurate single screening test using genetic or biochemical markers has been developed to determine which preschooler with recurrent wheezing will have asthma at school age, the asthma diagnosis still needs to be based on clinical predicted models or scores. The purpose of this review is to summarize the existing and most frequently used asthma predicting models, to discuss their advantages/disadvantages, and their accomplishment on all the necessary consecutive steps for any predictive model. Seven most popular asthma predictive models were reviewed (original API, Isle of Wight, PIAMA, modified API, ucAPI, APT Leicestersher, and ademAPI). Among these, the original API has a good positive LR~7.4 (increases the probability of a prediction of asthma by 2–7 times), and it is also simple: it only requires four clinical parameters and a peripheral blood sample for eosinophil count. It is thus an easy model to use in any rural or urban health care system. However, because its negative LR is not good, it cannot be used to rule out the development of asthma.

## Introduction

Wheezing is a sign associated with a highly heterogeneous set of conditions, with different pathogenesis and different outcomes during early life. Many epidemiological studies have shown, for example, that a large proportion of preschool children who wheeze, even recurrently, show remission of their symptoms either by age three, or during the school years (1, 2). Based on these findings, several early childhood wheezing phenotypes were described based on their natural history and associated risk factors (2). Since these phenotypes were described based on longitudinal data, they are of little use to the clinician when making therapeutic decisions affecting children whose future course is obviously unknown to him. Much more useful was a recent clinical trial showing that, although as a group, preschool children respond better to inhaled corticosteroids (ICS) than to montelukast, children who are not sensitized to aeroallergens or do not have peripheral blood eosinophilia respond equally well to both medicines (3). Thus, the clinician has now easily available tools to assess which preschool children are more likely to respond to ICS and in which children alternative approaches are still needed.

Of similar importance for the clinician, however, is the prognosis of the preschool child requiring medical attention for recurrent wheezing. Once atypical causes of wheezing are ruled out (e.g., cystic fibrosis, airway malformations, foreign bodies, etc.), parents are still eager to know if their child has “asthma,” which in their mind and in that of many clinicians is equated with a chronic disease that will persist and require treatment for extended periods during childhood and into adult life. Moreover, the great challenge toward the future is asthma prevention, and there is now solid data suggesting that in most cases of persistent asthma, the first symptoms most often develop during the school years (4), and it is during this age that the deficits in lung function growth, which are characteristic of the disease first appear (5–7). This has stimulated the search for indices or models that, combining different risk factors known to increase persistence of symptoms in young wheezing children, would allow identifying children more likely to have symptoms during the school years.

The purpose of this review is to compare and contrast different prediction tools developed during the last two decades. As stated, earlier, the role of these tools is not to facilitate attributing labels (“asthma”) that have little meaning in the face of the complexity and heterogeneity of wheezing conditions in this age group. At this moment, indices and models are also not useful for identifying children who will or will not respond to asthma therapy during the school years. Much more appropriate for this latter purpose are clinical trials such as the INFANT study (3) in which phenotypes and biomarkers are directly tested for their capacity to predict which children will respond to a certain therapy as compared to other therapies or placebo. The lain purpose of these indices and models is, as stated to predict outcomes and therefore, to allow clinicians to have meaningful discussions about prognosis with parents and caregivers, and to open the way to prevention studies that will be targeted to children at the highest risk of developing persistent symptoms later in life.

Clinical prediction rules (CPR) are decision-making clinical tools that use variables of medical history, physical examination, and simple laboratory tests to provide the probability of an outcome, prognosis, or likely response to treatment in an individual patient (8). At this point, it is very important to remark that the clinical usefulness of a diagnostic test is determined by the extent to which it helps to modify the pretest probability of occurrence of a certain diagnosis. For this purpose, the calculation and application of likelihood ratios (LR) is a very useful tool, reflecting the magnitude by which the pretest probability increases or decreases and thereby helping the physician rule out, confirm, or continue investigating a diagnosis with new tests (9). Therefore, LR, not the sensitivity or positive predicted value, is the best parameter reflecting the diagnostic accuracy of any diagnosis or prognosis model (10, 11).

It is important to remember that four consecutive steps are needed for prognostic or diagnostic prediction rules to become universal accepted for massive use. These steps are: development, validation, impact, and implementation (12).

## Predictive Models for Prognostic or Diagnostic Tools

### Step 1: Development

At this moment, at least seven predictive models or scores for asthma have been developed. The first model was the Asthma Predictive Index (API) or original API (13), followed by the PIAMA (14), Isle of Wight (15), modified API (mAPI) (16), University of Connecticut (ucAPI) (17), Asthma Prediction Tool (APT) from Leicester (18), and Asthma Detection and Monitoring (ademAPI) (19). The characteristics and predictors used in these models were shown in Table 1.

**Table 1:** Characteristics of currently available asthma predictive models.

	**Original API (13)**	**Isle of Wight (14)**	**PIAMA (15)**	**mAPI (16)**	**ucAPI (17)**	**APT (18)**	**ademAPI (19)**
Year publication	2000	2003	2009	2013	2014	2014	2015
Country	US	UK	Netherlands	US	US	UK	Netherlands
#children survey	1,246	1,034	2,171	289	589	1,998	202
Source population	General	High-risk	High-risk	High-risk	High-risk	High-risk	General
Age (y) asthma prediction	6, 8, 11, 13	10	7–8	6, 8, 11	7	6–8	6
Methods of building	Clinical index	Cumulate risk score	Logistic regression	Clinical index	Clinical index	LASSO regression	Logistic regression
#predictors used	5	4	8	5	5	10	8
**PREDICTORS**							
Age						✓	
Gender			✓			✓	
Wheezing frequency^*^	✓		✓	✓	✓	✓	✓
Parental history of asthma or allergy	✓	✓		✓	✓	✓	✓
Eczema	✓		✓	✓	✓	✓	✓
Rhinitis	✓	✓			✓		✓
Wheezing without colds	✓		✓	✓	✓	✓	✓
Blood eosinophilia	✓			✓			
Skin prick test		✓		✓	✓		
Specific IgE							✓
Chest infections		✓	✓				
Parental medication inhalation			✓				
Parental education			✓				
Post-term delivery			✓				
Activity disturbance						✓	
Shortness of breath						✓	
Exercise-related wheeze/cough						✓	
Aeroallergen-related wheeze/cough						✓	
EBC biomarkers							✓
VOCs							✓
Gene expression							✓

* *As enter criteria for stringent API, mAPI, ucAPI, and adem API. API, asthma predictive index; APT, asthma predictive tool; PIAMA, Prevention and Incidence of Asthma and Mite Allergy; mAPI, modified API; ucAPI, University of Cincinnati-API; ademAPI, Asthma Detection and Monitoring-API; EBC, exhaled breath condensate; Ig, immunoglobulin; VOCs, exhaled volatile organic compounds*.

In clinical prediction rules, each score level represents a different LR. Calculation is based on a proportion, in which we consider in the numerator, the proportion of patients with the condition with the given score level, and in the denominator, the proportion of patients without the condition with the given score level (9). For the purpose of simplifying, scores are sometimes dichotomized into positive and negative, i.e., scores are collapsed into two categories yielding LR considered as positive or negative, when given group of predictors are present or absent.

Regarding prediction of asthma, the positive LR is the probability of a child with active asthma to have been classified as being at risk divided by the probability of a child without active asthma to have been classified as being at risk. The negative LR is the probability of a child with active asthma to have been classified as not being at risk divided by the probability of a child without active asthma to have been classified as not being at risk. Traditionally the negative LRs < 0.1 and positive LRs > 10 are considered to be conclusive, whilst LRs in the range of 0.2–5 are of limited usefulness. However, beside the number, it is more important how big the change between the pre-test probabilities and the post-test probability is (9).

**Figure 1:**
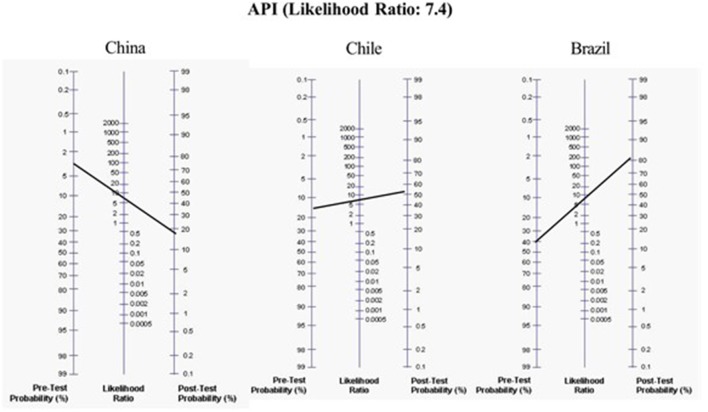
Application of the original positive Asthma predictive index (API) in hypothetical different scenarios with a low, moderate, or high-risk population of having asthma at school age.

The LRs of these different prediction models for assessing the development of asthma at school age were: original stringent API (13) (+LR = 7.43 and –LR = 0.75, for asthma at age 6 years); Isle of Wight (14) (+LR = 3.41 and –LR = 0.56, for asthma at age 10 years); PIAMA (15) (score ≥ 20, +LR = 2.5 and –LR = 0.53, for asthma at age 7–8 years); mAPI (16) (+LR = 21 and –LR = 0.84, for asthma at age 6 years); ucAPI (17) (+LR = 7.5 and –LR = 0.6, for asthma at age 7 years); APT (18) (+LR = 2.5 and –LR = 0.4, for asthma at 6–8 years); and the adem-API (19) (+LR = 8.8 and –LR = 0.13, for asthma at age 6 years) (Table 2). For example, to determine the risk of asthma in preschoolers from countries with high (e.g., Brazil), medium (e.g., Chile), and low (e.g., China) asthma prevalence, using the positive original stringent API, the probability for developing asthma increases by 2, 4, and 7 times (the pretest probability of asthma moves from 40 to 80%, from 14 to 62%, and from 3 to 21%, respectively) (20) (Figure 1). However, since their negative LR is not good, it cannot be used to rule out the probability for the development of asthma.

**Table 2:** Performance measures among current available asthma predictive models.

**Asthma predictive model**	**Sensitivity**	**Specificity**	**Positive**	**Negative**	**Positive**	**Negative**
			**PV**	**PV**	**LR**	**LR**
(13) Original API (stringent index)						
At 6 years	28	96	48	92	7.43	0.75
At 8 years	16	97	44	88	4.9	0.86
At 11 years	15	96	42	86	3.9	0.88
At 13 years	16	97	52	84	4.9	0.9
(14) Isle of Wight (score strata ≥3)						
At 10 years	53	85	68	74	3.41	0.56
(15) PIAMA (cutoff ≥ 20)						
At 7–8 years	60	76	23	94	2.5	0.53
(16) mAPI (from year 3)						
At 6 years	17	99	72	9	21	0.84
At 8 years	19	100	87	9	55	0.83
At 11 years	19	99	70	9	19	0.82
(17) ucAPI At 7 years	44	94	60	89	7.5	0.6
(18) APT At 6–8 years	72	71	49	86	2.5	0.4
(19) ademAPI^*^ At 6 years	88	90	90	89	8.8	0.13

* *Final model: API plus 9/17 volatile organic components and 3/31 gene expression markers. API, asthma predictive index; APT, asthma predictive tool; PV, predictive value; LR, likehood ratio; Positive LR, sensitivity/1-specificity; Negative LR, 1-sensitivity/specificity*.

Several important issues need to be addressed to determine the internal validity of the optimal prediction model. Among these seven models, only the original API (13) is relatively generalizable since it was developed in an unselected ethnically diverse birth cohort. The Isle of Wight (14) and PIAMA (15) included patients with respiratory tract/recurrent chest infections among their many criteria which could misrepresent the reporting of episodes of recurrent wheezing. The PIAMA (15) is more laborious to determine because the many criteria used have different weights, in addition, its generalizability may be reduced since it includes health beliefs and socioeconomic information that may vary between ethnicities and countries. The ucAPI (17) even though it was the first one that used an objectively confirmed asthma (change in FEV1 of ≥ 12% post bronchodilator or a methacholine challenge test PC_20_ ≤ 4 mg/ml), it was developed in a high-risk cohort. Similarly, the mAPI initially proposed by expert opinion was recently also developed in a high-risk of atopy birth cohort (16). Moreover, it is well-known that peripheral blood eosinophilia is a better predictor of remission of asthma, than specific IgE or skin prick test (21, 22). Therefore, predictive indices that use peripheral blood eosinophilia (a cheaper and worldwide common test) instead of specific or total IgE or skin prick test, will be more useful for predicting asthma. In addition, in the literature (23) recently started the debate about the cutoff for specific IgE (> 0.35 kUa/L) or positive skin test (≥3 mm) generally considered as indices of sensitization, being arbitrary and having not been defined by a scientific study; moreover, they depend on age and sex (23). Therefore, the incorporation of specific IgE or positive skin test in any predictive model without taking these consideration are questionable. The APT (18) tool was developed in high risk population (children came from health care visits because of respiratory problems) and incorporated age, gender, and eight clinical wheezing/atopy characteristics.

The latest model developed was the ademAPI (19). It included a prospective case-control study, adding to original API (but replacing eosinophilia by specific IgE) the following parameters: 17 exhaled volatile organic compounds (collected and detected by gas chromatography–time of flight–mass spectrometry), 10 exhaled breath condensate biomarkers (cytokines and chemokines analyzed with a multiplex immunoassay), 31 genes expression, and lung function measured by airway resistance using MicroRint^®^. This model is maybe the most complete and sophisticated model, but although it reaches the best positive and negative LRs (8.8 and 0.13, respectively), the possibility to be used massively in any healthy setting worldwide is very unlikely due to the high costly and sophisticated predictors included. Moreover, the increase of positive LR from 7.4 (original API) to 8.8 (ademAPI) might not justify all the sophisticate/expensive biomarkers used in the later tool.

### Step 2: Validation

After developing any prognosis model the next step is the validation, i.e., “testing the accuracy of the prediction rule in patients that were not included in the development study. Temporal, geographical, and domain validations can be distinguished. If necessary, the prediction rule can be updated, by combining the information captured in the rule (development study) and the data of the new patients (validation study)” (12).

At this moment, only the original API, PIAMA, and APT models were validated in new populations. The original API was validated in three different independent large cohorts and one small high-risk cohort. In the general population of Leicester Cohort from Switzerland (24) (*n* = 3,392), the performance of API to predict asthma at 7 years was LR (+) 5.3 and LR (–) 0.7 for the stringent API, and LR (+) 2.9 and LR (–) 0.5 for the loose API; at 10 years the figure was similar. In the high risk wheezer population (*n* = 1,573) of the PIAMA (15) cohort, the API performance to predict asthma at 7.8 years was LR (+) 5.3 for the stringent API and LR (+) 2.8 for the loose API. And in a nested case control study (*n* = 616) from a birth cohort in Norway (25) the stringent API for asthma at 10 years. was LR (+) 3.3 LR (–) 0.5. In a small study in Colombian recurrent wheezer preschoolers (*n* = 130) from outpatient clinic the API performance for predict asthma at 5–6 years was LR (+) 2.1 for stringent and 1.1 for the loose API (26). It is important to note that a different combination of API criteria was used in two validated studies (15, 24) that made the interpretation difficult.

In contrast, the PIAMA was validated in a high-risk population (*n* = 2,877) the Dutch R-Generation (27) where their performance for asthma at 6 years was AUC = 0.74; and in the small Colombian study where their LR (+) was 2.6 for asthma at 5–6 years (26). Also, the PIAMA score used in the Dutch study was not identical to the one use in the developed study. Finally, the APT score was validated in only a small high-risk population German study (28) (*n* = 140) where their performance for predicted asthma at 8 years was LR (+) 2.6, LR (–) 0.3, and AUC = 0.83.

### Step 3: Impact

The next step is to determine the impact of the CPR, i.e., “determining whether a validated prediction rule is used by physicians, changes therapeutic decisions, improves clinically relevant process parameters, improves patient outcome or reduces costs” (12). The ideal design for an impact study, is a randomized controlled study, in which the “intervention” group is randomized to use the CPR and the control group to use standard or usual judgement. Only a limited number of CPR worldwide have completed this step, probably because of the big methodological challenge and high costs of such studies for CPRs.

At this time, none of the prediction models has moved forward to this stage. Therefore, there is little evidence to support impact and implementation. Nevertheless, at this moment the original stringent API has been the most common predicted model tested worldwide in different non-randomized studies, the other was the mAPI. For example, Wi et al. (29) performed a cross-sectional study on 105 American children (5.8 ± 1.5 years. old) assessing performance of “retrospective” API against Predetermined Asthma Criteria, showing that the agreement rate and kappa between those were 89.5% and 0.66 (*p* < 0.01). This could suggest that application of API to retrospective studies for ascertaining asthma status is suitable. Similarly, a case-control study (30) on 202 Chilean children aged 6–7 years showed that those with current asthma had a positive API when they already were preschoolers [OR = 84.3 (24–436)]. In a case-control study (31) on 113 Brazilian children (aged 6–24 months) comparing recurrent wheezing vs. controls, the positive stringent API remained an important risk factor for recurrent wheezing [aOR = 5.57 [2.23–7.96], *p* < 0.001]. A study (32) on 529 Turkish school children with history of recurrent wheezing, reported that those with negative API in the past, had significantly shortened wheezing duration.

A recent cross-sectional study (33), nested in a US birth cohort, was done to develop and validate a natural language processing (NLP) algorithm to identify patients that meet the original API criteria. Asthma status ascertained by manual chart review based on API criteria served as gold standard. NLP-API was developed on a training cohort (*n* = 87) and validated on a test cohort (*n* = 427, median age of 5.3 years). The NLP-API predicted asthma with sensitivity 86%, specificity 98%, positive PV 88%, negative PV 98%. NLP-API was able to ascertain asthma status in children mining from electronic health records and has a potential to enhance asthma care and research through population management and large-scale studies when identifying children who meet API criteria.

Also, the original API was used to explore the effect of a smart nebulizing device on the rate of adherence to ICS in Chinese preschoolers with positive API (*n* = 65), showing a significant improve in the rate of adherence, and reducing the frequency of emergency visits and respiratory infections, as well as the usage of antibiotics or systemic steroids (34). A recent study (35) in Chinese Han preschoolers (*n* = 385) reported association of four-gene model consisting of *IL13* rs20541, *IL4* rs2243250, *ADRB2* rs1042713, and *FCER1B* rs569108 with the original API [OR = 4.08, *p* < 0.0001; and OR = 2.36, *p* < 0.0001, for stringent and loose API, respectively].

Also, the original stringent API was compared and correlated with surrogated markers of airway inflammation using two non-invasive tests, i.e., fractional exhaled nitric oxide (FeNO) and exhaled breath condensate (EBC) pH. In a study on 391 Switzerland preschoolers (age 3–47 mo), FeNO was significantly higher in those with positive stringent API than positive loose API or recurrent cough without history of wheeze controls (36). Also, in a study on 32 Spanish infants (median age of 12 mo) with recurrent wheezing, those with positive API had significant higher FeNO levels than those with negative API (37). Similarly, a study on 52 Argentinian preschoolers (aged 5–36 mo) showed a significantly higher levels of FeNO in the positive API group than in those with negative API (38). Finally, a study on 191 German preschoolers (median age 4.4 years) reported that reduced EBC pH value combined with API improved the chance to identify asymptomatic children at high risk of asthma (AUC = 0.88 for positive API vs. AUC = 0.94 for positive API plus pH) (39).

Other inflammatory biomarkers e.g., periostin, CC16 and YK-40, were also tested in population with positive and negative original API. A study done in 48 Chilean preschoolers (aged 24–71 mo) reported no significant differences in serum periostin levels for those with positive API and negative API; and no significant correlation between serum periostin levels and peripheral blood eosinophils (40). Also, no difference in level of serum CC16 levels for preschoolers with a positive API and negative API were found in the same population (41). A correlation between serum CC16 levels and age was found [*r* = 0.36 [0.07–0.59], *p* = 0.01], but not between serum CC16 levels and peripheral blood eosinophils. Similarly, a recent study done in 98 Turkish preschoolers showed that periostin and angiopoietin serum levels were similar between positive and negative mAPI (42). Finally, a longitudinal Sweden study of 156 preschoolers with recurrent wheeze and 101 healthy controls, showed that YKL-40 levels were elevated during acute wheeze exacerbation in positive and negative API (maybe related with current neutrophilic inflammation) compared to controls, but not at 3 and 12 month follow-up after the acute exacerbation (43).

The API was also compared and correlated with airway inflammation in bronchial biopsies. A study on endobronchial biopsies obtained from 30 Czech preschoolers (median age 13.5 mo) who underwent flexible bronchoscopy for various clinical reasons found significant difference in the thickness of the basement membrane, subepithelial deposition of laminin, and collagen IV in the basement membrane between children predisposed to asthma-by positive API- and control group (44).

Since there were some criticism about the value of the original API, some studies were performed changing or adding new biomarkers in order to improve the API performance for predicting asthma at 6 years of age. For example, on 191 Germany preschoolers (median age 4.4 years), when adding an exhaled breath condensate pH to the original stringent API, the positive LR was lower compared to the original stringent API (LR (+) 5.88 vs. 7.43, respectively) (39). Similarly, replacing FeNO instead of blood eosinophil determination from the original API in 391 Swiss preschoolers (aged 3–47 mo) results in lower positive LR (1.99) for predicting asthma at age of 4 years than original stringent API (45). In other study, interleukin-1-receptor-like 1 or sST2 (a well-replicated asthma-gene and associates with eosinophilia) was compared to the API in 202 Dutch wheezing children and 50 healthy controls reporting that serum sST2 levels at 2–3 years could not distinguish which of the preschoolers developed asthma at school age; consequently, serum sST2 did not significantly add to the prediction of asthma diagnosis than the used of API (API alone vs. API+sST2: AUC = 0.60, *p* = 0.02 vs. AUC = 0.57, *p* = 0.12, respectively) (46).

### Step 4: Implementation

Finally, the last step is the implementation, i.e., “actual dissemination of the diagnostic or prognostic prediction rule in daily practice to guide physicians with their patient management” (12). Although, like formerly exposed, none of the CPR has completed the impact step, the original API and mAPI are the only asthma prediction models that have been implemented worldwide over the years (Table 3).

**Table 3:** Steps for develop a prognostic or diagnostic prediction model (12).

	**Development**	**Validation**	**Impact**	**Implementation**
Original API	✓ (13)	✓ (15, 24–26)		✓ (47–56)
Isle of Wight	✓ (14)			
PIAMA	✓ (15)	✓ (26, 27)		
mAPI	✓ (16)			✓ (3, 55–62)
ucAPI	✓ (17)			
APT	✓ (18)	✓ (28)		
ademAPI	✓ (19)			

For example, the API has been used to explore the airway lung function in young asthmatic children. Using the tidal rapid thoraco-abdominal compression and raised volume technique, 50 Portuguese recurrent wheezing children aged 8–20 mo were compared to 30 controls, showing a significant lower z scores for FVC and FEF_25−75_ in those with positive than those with negative API (47). Later, a study of thoraco-abdominal compression technique on 91 recurrent wheezing Spanish preschoolers (aged up to 24 mo) showed that those with positive API had a lower Vmax FRC Z-score than negative API (48). A case-control study (49) on 108 Chilean recurrent wheezing preschoolers (aged 24–72 mo) showed no differences in basal lung function and post-bronchodilator response to salbutamol (by IOS or spirometry) between positive and negative API preschoolers. However, positive API preschoolers with ICS had significantly higher central basal airway resistance (RA at 20 Hz) and higher post-BD response (% change in FEF_25−75_ and FEV_0.5_) than those positive API without ICS; suggesting that preschoolers with positive API and ICS use may have some airway dysfunction (49). Recently, a Finish longitudinal study (50) about lung function assessed with IOS measured at preschool age (*n* = 255) and at adolescence, showed that abnormal baseline values (poor R5) during preschool were significantly associated with: low lung function, need for asthma medication, and asthma symptoms during adolescence. And a positive API at preschool age was associated with asthma symptoms (OR: 13.7 [1.4–147.1]) and need for asthma medication (OR: 14.6 [1.4–147.1]), but not with abnormal lung function at adolescence (50).

The mAPI has been also used to explore airway lung function. A study to assess whether the IOS has a diagnostic value to predict the mAPI in 115 Turkish preschoolers (median age 39 mo) with recurrent wheezing showed a significant improvement in the rate R5-R20% in children with positive mAPI compared to negative mAPI. The R5–R20% levels >14.4 had a sensitivity of 75% and specificity of 53% for predicting a positive mAPI [AUC: 0.656, *p* = 0.003] (57). In 34 Finnish children (aged 3–7 years) with recurrent wheezing measured by impedance pneumography at home during sleep, those with positive mAPI had significantly lower min curve shape correlation and minimum noise limit than negative mAPI, indicating a stronger change in flow profile shape and momentarily lowered chaoticity (58). Recently, the same group reported that children with positive mAPI presented reduced sympathvoagal balance, suggesting that high-risk of developing asthma might be related with a lack of adaptability of parasympathetic nervous system (59).

The original API has been also used in studies aimed to correlate with some nutrients. A Turkish study of 186 infants with recurrent wheezing compared with 118 healthy control peers, showed that those recurrent wheezing with negative API had significantly lower levels of vitamin D than those with positive API, and both had lower levels than controls (51). Similarly, a case-control study in *n* = 148 Turkish children (mean age 20.7 mo) showed lower serum levels of vitamin D and zinc, and higher Cu and Cu/Zn ratio in recurrent wheezing with positive API compared to those with negative API (52).

Finally, only the original API and mAPI has been used as recruitment tools in randomized control trials (RCTs). The API was used in two RCTs comparing ICS vs. placebo (53) and ICS vs. montelukast (54); and mAPI in four RCTs comparing ICS vs. placebo (60) and ICS vs. montelukast (3, 61, 62). Also, the original stringent API was endorsed by two asthma guidelines (55, 56), and the mAPI by one (55). A positive original API and mAPI preschool wheezer was proposed as one endotype, e.g., a Th2 mechanism and good responder to daily ICS, under the asthma syndrome umbrella (63). Two recent systematic reviews identified other prediction models for asthma but with considerable limitations (64, 65). A critical appraisal of asthma predictive models using the CHARMS checklist, revealed that original API, ATP, and PIAMA have low-moderate risk of bias (65). Finally, “several major strengths of the original API are in the design, with a simple set of equally weighted criteria with a binary scoring system: the criteria are either met or not met, allows easy implementation (only requires clinical parameters plus a peripheral blood sample for eosinophils count) and interpretation of the API” (66) in all type of rural and urban health services setting worldwide.

## Conclusion

Among all the prediction models for asthma prognosis, the original API is in most use because it derives from unselected multiethnic population, is simple to recollect, cheaper, little invasive (e.g., peripheral blood count) and has been validated in external populations. It is a very promising predictive tool and the positive original stringent API should be able to be used by clinicians worldwide in any health setting, to identify at-risk children and educate parents on the importance of asthma maintenance therapy and treatment of flares. Its major strength is its positive LR~7.4 that allows an important effect on post-test probability of disease, improving significantly by 2–7 times the probability (Figure 1). But since its negative LR is not very low enough, it cannot be used to rule out the development of asthma.

## Author Contributions

JC-R contributed to the study concept, literature search, data collection, and manuscript writing. LC and FM contributed to review the manuscript and incorporated significant thought.

### Conflict of Interest Statement

The authors declare that the research was conducted in the absence of any commercial or financial relationships that could be construed as a potential conflict of interest.
